# The Role of Type I and Type II NKT Cells in Materno-Fetal Immunity

**DOI:** 10.3390/biomedicines9121901

**Published:** 2021-12-14

**Authors:** Eva Miko, Aliz Barakonyi, Matyas Meggyes, Laszlo Szereday

**Affiliations:** 1 Department of Medical Microbiology and Immunology, Medical School, University of Pécs, 12 Szigeti Street, 7624 Pécs, Hungary; barakonyi.aliz@pte.hu (A.B.); meggyes.matyas@pte.hu (M.M.); szereday.laszlo@pte.hu (L.S.); 2 Janos Szentagothai Research Centre, 20 Ifjusag Street, 7624 Pécs, Hungary; 3 National Laboratory for Human Reproduction, University of Pécs, 7624 Pécs, Hungary

**Keywords:** type I NKT, type II NKT, reproductive immunology, materno-fetal interface, implantation failure, pregnancy loss, mouse, human

## Abstract

NKT cells represent a small but significant immune cell population as being a part of and bridging innate and adaptive immunity. Their ability to exert strong immune responses via cytotoxicity and cytokine secretion makes them significant immune effectors. Since pregnancy requires unconventional maternal immunity with a tolerogenic phenotype, investigation of the possible role of NKT cells in materno-fetal immune tolerance mechanisms is of particular importance. This review aims to summarize and organize the findings of previous studies in this field. Data and information about NKT cells from mice and humans will be presented, focusing on NKT cells characteristics during normal pregnancy in the periphery and at the materno-fetal interface and their possible involvement in female reproductive failure and pregnancy complications with an immunological background.

## 1. Introduction

The hierarchic and yet complementary cooperation between innate and adaptive immunity during a classical immune response to combat foreign substances is a basic and well-known phenomenon in immunology, ensuring both rapid-acting and developing long-term immunity as well. However, these two distinct types of immune functions must be somehow integrated, modulated, and controlled.

**Figure 1:**
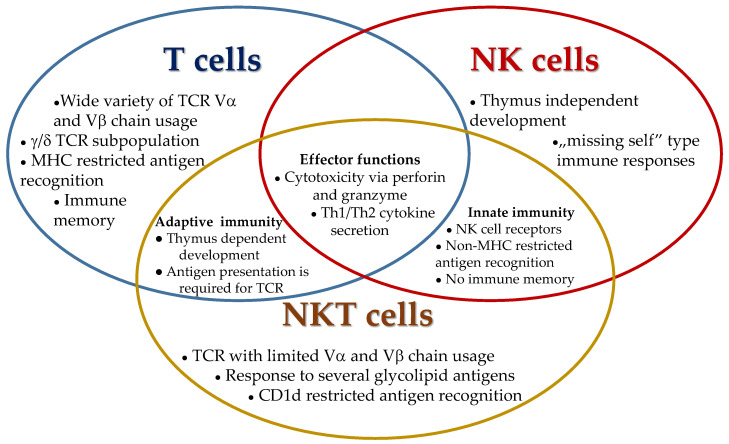
Shared and unique immunological characteristics of T, NK, and NKT cells.

NKT cells represent one of the bridging components by sharing properties of both natural killer (NK) cells and T lymphocytes. The innate-like characteristics of NKT cells are based on the expression of activating and inhibitory NK cell receptors operating according to self-antigen expression contributing thereby to protection from autoimmunity and exaggerated immune responses (Figure 1) [1,2]. Upon TCR expression, there are two different subsets of NKT cells: type I NKT or invariant (iNKT) cells, which carry a semi-invariant Vα14-Jα18 with Vβ2, 7, 8 TCR in mice and Vα24-Jα18 with Vb11 in humans; type II NKT cells with varying TCRs on their cell surface (Table 1) [3,4,5].

After their development in the thymus, most NKT cells home at different body sites and tissues where they long-time reside [6,7]. The NKT cell population can recognize various lipid-based antigens of exogenous (e.g., bacterial) or endogenous origin presented by an MHC I-like molecule, CD1d, on antigen-presenting cells [8,9,10,11]. For example, iNKT cells generate a robust cytokine response after stimulation with an agonist, α-galactosylceramide (αGalCer), derived from a marine sponge [12].

In comparison to the murine variant, humans have fewer iNKT cells and show a wider variation in the amount of circulating iNKT cells. Tissue-resident iNKT cells were identified in the liver, spleen, lungs, intestine, lymph nodes, and skin. One possible activation of these lymphocytes occurs via engagement with their invariant TCR. However, type I NKT cells are known for their potential to respond very quickly to danger and stress signals and pro-inflammatory cytokines. Upon activation, they immediately transform to significant effectors in an innate-like manner releasing large amounts of T helper 1 (Th1)- and/or Th2-type cytokines and chemokines as well [13]. iNKT cells are also responsible for the transactivation of many lymphocytes and leukocyte subpopulations: they promote T cell activation and differentiation, macrophage polarization, NK, dendritic cell and B cell activation, and neutrophil recruitment as well [13]. Due to these abilities of iNKT cells, they not only bridge but even modulate both innate and adaptive immune responses in a considerable way. Therefore, they are important factors in determining host immune responses to microbes or malignant cells, for example. Subpopulations of iNKT cells are similar to T cell subsets: Th1-like, Th2-like, and Th17-like iNKT cells have been identified as major cell subsets according to their cytokine expression pattern. In addition, there are two minor subtypes with T follicular helper-like function and IL-10-mediated regulatory functions [14].

Type II NKT cells are the major NKT subset in humans with a higher frequency than type I NKT cells [14]. In contrast to iNKT cells, type II NKT cells are not reactive to α-GalCer; instead, they are thought to respond to a diverse repertoire of lipid antigens, such as self-lipids or ligands of microbial origin [3,8,13]. Unlike in the case of type I NKT cells, activation of type II NKT cells is not fully understood, but besides activation by TCR-engagement, TCR independent ways, such as via pro-inflammatory cytokines, have been demonstrated [11]. Rapid-acting after activation is also a major characteristic of type II NKT cells, with the secretion of a wide range of cytokines and transactivation of other immune cells as described in iNKT cells [5,15]. Subgrouping of type II NKT cells is still not apparent. Despite less specific information about type II NKT cells, there is remarkable research on the possible involvement of this cell subset in different pathologic conditions such as: multiple sclerosis, type I diabetes mellitus, ulcerative colitis, obesity, liver diseases, and infectious diseases [15,16,17].

Pregnancy represents an exceptional challenge for the maternal immune system. On the one hand, immune tolerance mechanisms are required for the semi-allogeneic fetus, for its implantation and placentation processes both on a local and on a systemic level as well [18]. On the other hand, even during pregnancy and despite the materno-fetal immune tolerance, the maternal immune system has to be prepared for possible and potential immunological threats, such as infections [19]. Therefore, NKT cells could be important mediators by balancing maternal immune responses during pregnancy.

This review focuses on the possible role of NKT cells in physiological materno-fetal immune tolerance mechanisms and their changes observed in pregnancy complications in both mice and humans.

## 2. Immunological Characteristics of Murine Pregnancy

Translational research of maternal immunological changes during murine pregnancy is widely accepted in the field of reproductive immunology since considerable similarities in both implantation and placentation have been found and proven [20,21,22]. Both species belong to the monophyletic group of supraprimates with characteristic hemochorial placentation, where trophoblast invasion into the maternal uterus occurs [22].

Since the developing fetus does not come into direct contact with maternal tissues during the whole period of pregnancy, the trophoblast of fetal origin will represent the embryonal site at the materno-fetal interface [23]. As the initial steps of pregnancy, implantation and placentation take place almost simultaneously and the earliest events of maternal immunological recognition of the semiallogeneic fetus start within this period. The short period of time where the endometrium is receptive to an adequately developed embryo is called the window of implantation. As the preparation for pregnancy, the endometrium is transformed into a highly specialized, nutrient-rich decidual tissue under the influence of progesterone and induced by the implantation itself [24]. Uterine lymphocyte enrichment enables immunological recognition of the fetal site from the beginning [22,23].

The developmental stage of the embryo able to implant is the blastocyst, where a trophoblast layer has been already differentiated to become a placenta later as an outside layer of fetal cells. Class 1a major histocompatibility antigen (MHC) expression by fetal trophoblast was demonstrated with the occurrence of a small amount of paternally derived foreign antigens, but there is also a lack of definitive information about it [23]. Direct contact and possible immune interaction with decidual lymphocytes are thought to take place between fetal spongiotrophoblast and decidua basalis in murine placenta. Maternally and paternally derived MHC antigens might be recognized by NK cells, which are found to be the dominant lymphocyte subpopulation in the murine uterus [25]. Uterine NK cells express receptors that can directly interact with MHC antigens on the trophoblast leading to the unique physiological state of immune tolerance, which is characterized by a fine balance of dominant Th2 type immunity with significantly reduced pro-inflammatory responses under the tight control of immune regulatory mechanisms locally [23]. Although murine spongiotrophoblast cells do invade the decidua, the invasion occurs later, in the second half of gestation, in a rather discrete manner [20,22].

## 3. NKT Cells in Murine Pregnancy

The possible involvement of NKT cells in materno-fetal immune responses was demonstrated by investigations verifying the local presence of these cell populations and their ligands at the materno-fetal interface. A remarkable and temporally increase in decidual NKT cells was observed at the time of peri-implantation, which decreased later to the level of NKT cells found in the spleen [26]. Interestingly, decidual NKT cells of early pregnancy show a unique phenotype being double negative for CD4 and CD8 expression and carrying dominantly the Vβ3 chain and less the Vβ8 chain with the constitutively expressed Vα14 [26]. Others report a predominant Vβ7 expression [27]. Placental expression of CD1 suggests a possible interaction between decidual NKT cells and ligands presented by CD1 [26]. Since decidual NKT expression is not altered in CD1 KO mice but is greatly reduced in β2microglobulin-deficient animals, the development of uterine NKT cells is regulated by class I molecules other than CD1 may be of paternal origin [28]. This observation and the special phenotype of the decidual NKT cell population led to the hypothesis of local expansion and de novo development of decidual NKT cells in mice [28]. According to another study, where a single physiological exposure to semen by natural insemination led to the activation of T and NKT lymphocytes in paraaortic lymph nodes, homing of these activated cells to the uterus may also contribute to the local expansion of decidual NKT cells in early pregnancy [29].

The functionality of the cells was tested with α-GalCer administration to pregnant mice with the result of the high frequency of fetal resorption [27]. α-GalCer injections at different days of early pregnancy and even before conception revealed that the treatment had no effect on implantation success but inhibited further embryo development [27]. NKT cell-induced embryo resorption was found to be mediated by IFN-gamma, TNF-α production, and by perforin-mediated killing in this setting [27]. Moreover, two distinct mechanisms of pregnancy loss induced by α-GalCer have been demonstrated: perforin-dependent events at early gestation and a perforin-independent, cytokine-dominated mechanisms after midgestation [30]. In contrast to results from other organs, such as the liver, besides activation of uterine NKT cells with α-GalCer, the stimulation not only limited apoptosis of the cells but also induced their further expansion [31]. The expansion of iNKT cells could also be observed when α-GalCer treatment was carried out later in pregnancy, inducing preterm birth and neonatal mortality [32]. Therefore, a high incidence of fetal deaths observed during maternal infections could be mediated at least partially by a pathogen-activated and expanded decidual NKT cell population. This hypothesis was tested in a series of experiments where the maternal infection was imitated by the LPS treatment of pregnant mice. LPS recognition by Toll-like receptors (TLRs) on dendritic cells (DCs) results in NKT activation through cytokine secretion [33,34]. In mice with iNKT deficiency, LPS-induced preterm birth was found to be reduced [33]. Depletion of iNKT cells resulted in a decreased expression of costimulatory molecules on DCs and a decline of decidual iNKT cells in a number of activities [33]. Adoptive transfer of decidual iNKT cells to deficient animals restored the inflammatory response to LPS treatment [35]. If LPS was administered to wild-type mice in early pregnancy, increased fetal resorption could be observed with an upregulation of activated, pro-inflammatory iNKT cells in the decidua [34]. These data underline the central role of decidual iNKT cells mediating inflammation during infection. In line with these findings, α-GalCer administration in late pregnancy-induced preterm birth or neonatal death with the decidual expansion of iNKT cells [32]. If α-GalCer treatment was carried out in early pregnancy, miscarriage, and a local increase in iNKT cells could be observed both in syngeneic as wells as in allogeneic matings with the demonstration that α-GalCer selectively activated DEC-205+ DCs and secreted IL-12 from these activated cells may stimulate iNKT cells [36]. Moreover, α-GalCer administration induced elevated serum levels of IFN-gamma and IL-12 in pregnant mice induced by iNKT cells [37]. The central role of IFN-gamma in pregnancy loss was clearly demonstrated by a series of adoptive cell transfers to α-GalCer-treated iNKT-deficient pregnant mice. Both wild-type as well as iNKT cells from IL-4-deficient mice could augment fetal resorption via adoptive transfer, while iNKT cells from IFN-gamma-deficient mice failed to induce pathologic changes in pregnancy [38]. Adoptive transfer of regulatory T cells in wild-type pregnant mice could efficiently reduce the rate of pregnancy loss due to α-GalCer stimulation, suggesting the potential of immunoregulatory mechanisms to restore tolerogenic immune responses [38].

## 4. Immunological Characteristics of Human Pregnancy

The chain of events leading to successful implantation and placentation ensuring healthy pregnancy from the reproductive, immunological point of view has been intensively studied and has been characterized very detailed. As described in murine pregnancy, humans show haemochorial placentation with a particularly invasive trophoblast invading not only the endometrium but the first third of the myometrium [22,23]. For endometrial receptivity, decidualization occurs in the late secretory phase of the menstrual cycle before implantation affecting the entire mucus layer [22].

The human trophoblast differentiates in the first weeks after successful implantation in two types: villous and extravillous trophoblast. Villous trophoblast will create floating villi in maternal blood with an outer layer of fused trophoblast cells called syncytiotrophoblast and a basal inner structure of cytotrophoblast. After the first trimester of pregnancy, the placental supply of the developing embryo should increase significantly, and the placenta needs to contact more maternal blood [39]. For this fetal requirement of enhanced nutrition, trophoblast initiates remodeling of maternal spiral arteries, responsible for delivering blood directly into the placental unit. Extravillous cytotrophoblasts acquire an invasive, tumor-like phenotype with the ability to penetrate maternal decidual spiral arteries and close them for the first trimester with trophoblast plugs when placental perfusion is minimal [39,40,41]. Later, when fetal growth accelerates, trophoblast plugs disappear, and the extravillous cytotrophoblast creates the second stage of extensive invasion and spiral artery remodeling in-depth [41]. At the second half of pregnancy, this deep placentation is complete, and maternal circulation can sufficiently supply the expanding placenta.

The invasive character of human placentation requires susceptive maternal tissues that are ready to provide access to trophoblast penetration. For limiting trophoblast invasiveness in maternal tissue in time and space, a unique phenomenon, maternal immunological recognition of invading trophoblast, is thought to be responsible. Infiltrating extravillous cytotrophoblast expresses a unique combination of MHCs/HLAs, namely HLA-C, -E, F, and-G [42]. Non-classical HLA-E, F, and G show restricted polymorphism with significant similarities between maternal and paternal antigens. Only HLA-C is polymorphic, meaning that the immunological foreign paternal site is represented by the small amount of paternally inherited HLA-C expression of extravillous cytotrophoblast at the materno-fetal interface. In the decidua, the invading trophoblasts come across maternal lymphocytes. Decidual lymphocytes enrichment predominantly by NK cells that express activating and inhibitory receptors and can recognize the exact combination of HLAs displayed by the invasive extravillous cytotrophoblast resulting in a possible immune recognition of fetal antigens and creating thereby a very special immune response pattern [39,40]. Penetration of trophoblast relies not only on the invasive character of invading cells but requires a permitted uptake of the recipient tissue in the form of tissue loosening. The immune recognition of foreign paternal HLA-C antigens by decidual NK cells results in the classical activation of pro-inflammatory immune responses, dominated by cytokines, such as IFN-γ. The emerging inflammation itself could create favorable conditions for trophoblast invasion through secondary tissue damage in situ where it develops. Limitation and control of the invasion are possible due to maternal immune tolerance mechanisms induced by the recognition of placental non-classical HLA-E, F, G antigens. It is of note, and it should be emphasized as well, that a developing maternal immune response results not only in the immune acceptance of the fetus but significantly contributes to successful implantation and placentation [41].

Besides immunological changes at the materno-fetal interface locally, there are also small but significant alterations of systemic immunity with Th2 orientation observed in the peripheral blood of healthy pregnant women [43,44,45].

## 5. NKT Cells at the Periphery in Human Pregnancy

Little is known about to what extent NKT cells contribute to maternal immunotolerance mechanisms at the systemic level. Regarding the ratio of NKT cells in the peripheral blood of women with early pregnancy, incoherent data exist depending on the investigated NKT cell type. Before the gestational age of 10 weeks, the ratio of peripheral iNKT cells was similar to those of nonpregnant subjects [44]. In contrast, the percentage of type II NKT cells increased, according to another study [46]. Both studies confirmed the capability of cytokine production (IFN-γ, IL-4) of iNKT/type II NKT cells [44,46]. In the third trimester, investigating type 1/2 immunity of type II NKT cells based on surface expression of different cytokine receptors, type II NKT cells with anti-inflammatory activity were significantly increased at the periphery suggesting the involvement of this innate cell population in materno-fetal immune crosstalk [45]. Increased peripheral type II NKT cells at the late stages of gestation may be partially hormonally induced since leptin treatment of nonpregnant lymphocytes in a dose comparable to hormone concentration during the second-third trimesters resulted in a greater size of the cell pool [47]. It is not clear whether these cells contribute to the enhanced Th1 cytokine levels observed after leptin treatment, a possibility that would contradict the previous finding with increased type II NKT cells of type 2 immune profile during the third trimester [45,47]. At the very end of pregnancy, spontaneous vaginal delivery was associated with a significantly lower absolute count of peripheral type II NKT cells compared to elective C-section presuming selective migration of type II NKT cells from the periphery to the decidua [48].

Unfortunately, besides this small number of studies with phenotypic characterizations of peripheral NKT cells in normal pregnancy leading to conflicting results functional investigation regarding cytokine profile, cytotoxicity is completely missing. Therefore, appreciation of the role of peripheral NKT cells in materno-fetal immunotolerance must be waited for.

## 6. NKT Cells at the Materno-Fetal Interface in Human

**Figure 2:**
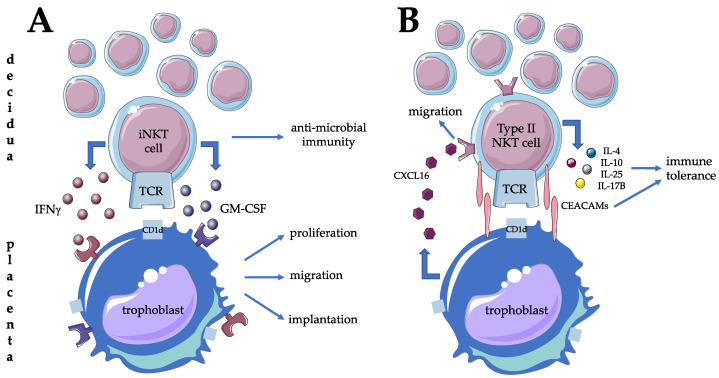
Possible contribution of type I (**A**) and type (**B**) NKT cells to materno-fetal tolerance mechanisms and successful implantation at the materno-fetal interface in humans.

In contrast to the periphery, the local presence and activity of iNKT cells and type II NKT cells have been investigated more in detail. First of all, CD1d expression was demonstrated, both on villous and extravillous trophoblast layers, suggesting the possibility of fetomaternal immune crosstalk through ligand expression from the fetal side [49,50,51]. Maternal decidual tissue from the first trimester showed significant enrichment of iNKT cells compared to the periphery with a striking bias toward pro-inflammatory activity with higher production of IFN-gamma and GM-CSF [44,49]. Since IFN-gamma is necessary for early decidual and placental development and trophoblast cells express GM-CSF receptors, iNKT cells could play a major role in establishing a favorable micro-environment during implantation and placentation (Figure 2A) [49]. Interestingly, type II NKT cells are found to be functionally distinctive. They also accumulate in the decidua but exert CD1d restricted anti-fetal all responses via cytokine secretion such as IL-4, IL-10, IL-25, and IL17B [52,53,54]. One possible mechanism leading to the observed Th2 phenotype of type II NKT cells is the inhibitory activity of cell surface CEACAM1 protein upon homotypic interaction with CEACAM1 expressed by the extravillous cytotrophoblast [55]. Investigating the migration of decidual type II NKT cells, CXCL16 expression by trophoblast is thought to be responsible for the retention of decidual type II NKT cells through interaction via their CXCR16 (Figure 2B) [56]. Increasing the adhesive capacity of type II NKT cells to endothelium can be observed even before pregnancy at the time of ovulation [57]. Besides recruitment, the decidual presence of NKT progenitors is another possibility for local enrichment of this cell population [58]. The possible contribution of decidual iNKT cells to local cellular events of term birth is controversial. A significantly higher proportion of this lymphocyte subpopulation was reported supporting this hypothesis [32,59], while others could not confirm it [60].

## 7. NKT Cells in Pregnancy Complications in Human

The largest amount of data comes from studies regarding the role of NKT cells in female infertility/recurrent spontaneous abortion (RSA). Recurrent pregnancy loss, also referred to as a recurrent miscarriage or habitual abortion, is historically defined as three consecutive pregnancy losses prior to 20 weeks from the last menstrual period. Epidemiological studies have revealed that 1% to 2% of women experience recurrent pregnancy loss [61]. However, the involvement of the iNKT cell population in the pathogenesis of miscarriage was investigated only in two studies; neither of them could reveal any association between the iNKT lymphocytes and the clinical syndrome [62,63]. In RSA patients, no significant changes were observed in the ratio of iNKT cells in the peripheral blood of the patients [62]. Focusing on possible alterations of decidual iNKT cells in sporadic miscarriage, there were no differences [63]. In the case of type II NKT cells, the situation is actually more difficult. Regarding changes of peripheral type II NKT cells in female infertility, recurrent implantation failure, and RSA, data are inconsistent and controversial. Considering type II NKT cell ratios in the peripheral blood of women with the mentioned pregnancy complications, no alteration, increase, and reduction were reported by different investigators. In one study investigating 10 women with three or more consecutive pregnancy losses, increased percentages of type II NKT cells in the peripheral blood were observed compared to normal early pregnancy and nonpregnant conditions [64]. Moreover, when focusing on the correlation of pregnancy success and peripheral type II NKT cell ratios, percentages of type II NKT cells more than 3.75% correlated negatively with pregnancy outcome [64]. In contrast to these findings, in two larger studies with RSA patients, a significantly lower percentage and absolute count of type II NKT lymphocytes were described than in healthy, nonpregnant controls [65,66]. However, no significant difference in the proportion of peripheral blood NKT cells and no association with pregnancy outcome between women with idiopathic recurrent pregnancy loss and healthy controls were also observed [67]. The predictive value of peripheral type II NKT cell ratio could not be confirmed as well when analyzing pregnancy success of women with immunologic infertility and/or pregnancy loss in the first trimester [68]. Investigation of decidual type II NKT cells in RSA has been the focus of only three studies, from that two failed to show changes of these cell populations locally in RSA patients [69,70], and one found a parallel increase in decidual type II NKT cells with the periphery in patients with RSA compared with normal early pregnancy [64]. Analyzing type II NKT cells from menstrual blood of infertile women, a significant reduction could be observed compared to healthy nonpregnant women [71]

Recurrent implantation failure refers to failure to achieve a clinical pregnancy after the transfer of at least four suitable-quality embryos in a minimum of three fresh or frozen cycles in a woman under the age of 40 years [72]. Analyzing the role of type II NKT lymphocytes in IVF success is not univocal. Both elevated [73], as well as decreased [74] and unchanged type II NKT cell ratios [75,76] were reported to be beneficial for successful implantation and pregnancy. Furthermore, a transient decrease in the immune checkpoint molecule, Tim-3 expression on type II NKT cells could be observed in the first week after successful IVF supporting the hypothesis of a favorable pro-inflammatory milieu for embryo implantation [75]. Data exist about the possible involvement of follicular fluid type II NKT cells in successful embryo implantation after the IVF procedure. According to one study, the relative distribution of type II NKT cells in the follicular fluid was significantly higher in women with unsuccessful embryo transfer than in those who conceived, but further studies are needed to explore the possible predictive value of this finding [76]. Besides these confusing and controversial data, three studies with large sample sizes support the proposition that intravenous immunoglobulin therapy improves pregnancy outcomes in selected patients with elevated type II NKT cell ratios [77,78,79].

Preterm birth is commonly defined as any birth before 37 weeks completed weeks of gestation [80]. In the case of preterm birth without chorioamnionitis, there could be an enhanced accumulation and aberrant activation of decidual iNKT cells as the initial event of the pregnancy complication as suggested by one group [81,82] while others could not confirm it [60].

Pre-eclampsia is one of the most severe pregnancy-specific syndromes, which affects 3–8% of all pregnancies. The definition of the disorder is gestational hypertension in previously normotensive women accompanied by one or more of the following new-onset conditions at or after 20 weeks of pregnancy: proteinuria, maternal organ dysfunction, uteroplacental dysfunction [83]. Pre-eclampsia is differentiated into early- (until 34 weeks) and late-onset (after 34 weeks) pre-eclampsia [84]. Development of early-onset pre-eclampsia is linked to inadequate invasion of the extravillous cytotrophoblast, which disturbs the remodeling of the maternal spiral arteries resulting in abnormal placentation and an oxidatively stressed small-sized placenta (poor placentation). It is thought that pre-eclampsia may be a form of maternal immune rejection of the semiallogen fetus. Clinical symptoms usually occur from week 20 because the abnormal placenta is not able to compensate for the continuously accelerating growth of the fetus after the 20 weeks of gestation [41]. Few but consistent data exist regarding the possible role of both iNKT and type II NKT cells in the pathogenesis of the inflammatory stage of pre-eclampsia. In preeclamptic women, NKT cells were found to exhibit a Th1-dominant profile with an altered NK cell inhibitory and activating receptor expression pattern contributing to the maternal systemic inflammatory response in the clinical phase of the disease [45,85].

## 8. Conclusions

Taking NKT cell characteristics into account, there is no doubt about the merit and significance of investigating NKT cells in materno-fetal immune tolerance. The local presence of both NKT cells and their ligands at the materno-fetal interface was shown in mice and humans as well suggesting possible interactions and immediate involvement in maternal immune responses toward the fetus. Although these findings and despite intensive research, there is still no better understanding and consistent concept of the actual role of these immune cell populations in materno-fetal immune responses. There are some possible explanations for controversial data in this field. One of them is that simultaneously investigation and identification of both NKT type I and type II lymphocytes is very unusual. Most studies are focusing only on one type of them, hampering comparison of the obtained results. Investigating NKT cells as a part of large-scale immunological studies could be another limiting factor for interpretation.

There are convincing data from several studies suggesting preserved pro-inflammatory activity of decidual NKT cells to mediate adequate immune responses in the case of a potential infection. According to this hypothesis, NKT cells could even represent a cell population with less contribution to immune tolerance. However, considering the fact that even during maternal tolerogenic immune responses, there should be a small but essential amount of inflammation, it could be at least partially provided by the NKT cell population.

## Figures and Tables

**Table 1:** Comparison of human type I and type II NKT cells.

HUMAN	Type I NKT Cells	Type II NKT Cells
Synonym	Invariant NKT cells (iNKT)	Non-invariant NKT cells
TCR chain usage	Vα24Jα18-Vβ11	Various
CD1d restriction	Yes	Yes
Proportion	~95%	~5%
α—GalCer reactivity	Yes	No
Cytokine expression	IFNγ, IL-4 (IL-3)	IFNγ, IL-4 (IL-13, IL-10)
NK receptor expression	Yes	Yes
Cytotoxicity	Yes	Yes

## Data Availability

Not applicable.
